# An effective method for determining necking and fracture strains of sheet metals

**DOI:** 10.1016/j.mex.2021.101234

**Published:** 2021-01-18

**Authors:** Ruiqiang Zhang, Zhusheng Shi, Zhutao Shao, Victoria A. Yardley, Jianguo Lin

**Affiliations:** Department of Mechanical Engineering, Imperial College London, London SW7 2AZ, UK

**Keywords:** Spatio-temporal method, Formability, Limit strain, Localised necking, Forming limit diagram

## Abstract

Biaxial tensile testing methods using cruciform specimens have been developed in the last few decades for the determination of forming limit diagrams (FLDs) and fracture forming limit diagrams (FFLDs) for sheet metals. One of the difficulties associated with this test geometry is the lack of a widely accepted method to determine the necking and fracture strains which are necessary to construct these diagrams. In this study, a novel spatio-temporal method has been proposed for the determination of necking and fracture strains. In the method, two rectangular zones: the base zone (BZ) and the reference zone (RZ) are selected at the location where fracture initiates. The zone RZ includes the zone BZ and both zones have the same side length in the direction parallel to the necking band but different side length in the perpendicular direction. By plotting the thickness reduction within RZ against that in BZ, the onset of localised necking can be determined by finding the intersection of the two straight lines fitted separately using the data in the initial and final stages of deformation. The corresponding limit strains are then determined using the strains within the zone BZ. The method has been successfully applied to uniaxial tensile tests on AA6082 and boron steel dog-bone specimens, and to equi-biaxial tensile tests on AA5754 cruciform specimens.

• Compared to widely used existing methods, the novel spatio-temporal method has greater simplicity, stability and accuracy with regard to the determination of localised necking strains.

• The spatio-temporal method has good potential to become a standard method for the determination of limit strains for sheet metals.

## Graphical abstract

**Figure:**
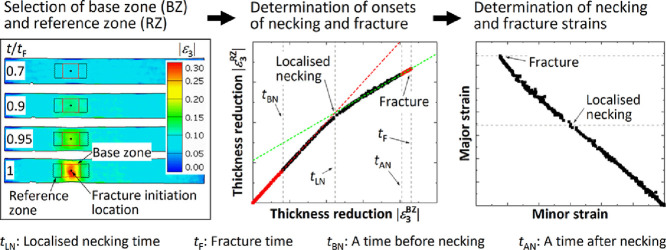

Specifications Table

**Table:** 

Subject Area:	Engineering
More specific subject area:	Formability evaluation for sheet metals
Method name:	Novel spatio-temporal method for limit strain determination
Name and reference of original method:	N/A
Resource availability:	N/A

## *Method details

 

## Motivation

Formability evaluation for sheet metals is essential for the development and optimisation of advanced forming technologies, especially for those at elevated temperatures (e.g. hot stamping). Forming limit diagrams (FLDs) and fracture forming limit diagrams (FFLDs) have been widely used as effective tools for the evaluation of formability. In FLDs, the limit strains at the onset of localised necking are presented in the space of major and minor strains, while in FFLDs, the limit strains at fracture are plotted on the same axes. Usually, FLDs are determined by using the Nakajima tests, and the limit strains at the onset of localised necking in the tested samples are measured using the cross-section method, as described in the international standard ISO 12004:2 2008 [1]. However, due to the complex forming and quenching process in hot stamping, it is extremely difficult to apply the Nakajima tests to determine FLDs under these conditions.

Biaxial tensile testing using cruciform specimens has great potential for the determination of FLDs and FFLDs under complex forming conditions. For this purpose, Lin et al. [2] developed a novel biaxial testing system, in which a patented link-mechanism rig has the capability of transferring an input uniaxial force to output biaxial forces to stretch cruciform specimens. Using this novel biaxial tensile testing system, Shao et al. [3,4] first attempted to determine FLDs of 1.5 mm thick AA6082 sheet under hot stamping conditions. They confirmed that the temperature in the gauge area of the cruciform specimens could be controlled accurately. In order to apply the biaxial testing method to boron steel under hot stamping conditions, Zhang et al. [5,6] successfully extended the application of the digital image correlation (DIC) technique to measure strain fields, and Zhang et al. [7] also developed a new heating method for cruciform specimens to give a more uniform temperature distribution within the gauge area. However, a widely accepted method for limit strain determination in the tested cruciform specimens is still lacking, which severely restricts the application of the biaxial tensile testing method.

## Spatio-temporal method

### Method description

**Fig. 1:**
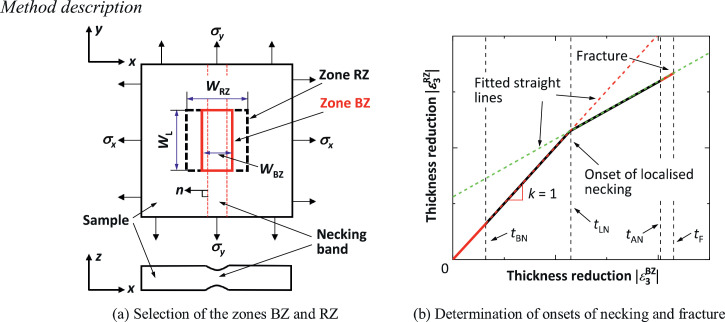
Schematic showing determination of necking and fracture strains using the spatio-temporal method. Note that zone RZ includes zone BZ, and both zones RZ and BZ have the same length $W_{L}$ of the sides parallel to necking band.

A spatio-temporal method has been proposed and developed for the determination of limit strains at the onset of localised necking and fracture for sheet metals. In this method, two rectangular zones, named the base zone (BZ) and the reference zone (RZ), are selected at the location where fracture initiates in the tested samples, as shown in Fig. 1(a). The zone RZ includes the zone BZ, and at the start of the deformation, both zones have the same initial side length $W_{L}$ in the direction parallel to the necking band, while they have different initial side lengths in the direction normal to the necking band, i.e. $W_{\text{RZ}}$ and $W_{\text{BZ}}$ (where $W_{\text{RZ}}>W_{\text{BZ}}$). The onset of localised necking is determined by plotting the average thickness reduction within the zone RZ ($|\varepsilon_{3}^{\text{RZ}}|$) against that in the zone BZ ($|\varepsilon_{3}^{\text{BZ}}|$) throughout the deformation, as shown in Fig. 1(b). In this study, the thickness reduction is derived based on the volume conservation assumption, by measuring the major and minor strains using the DIC technique [8], [9], [10]. It should be noted that the volume conservation assumption may break down after severe necking, but further research is needed to clarify this effect. In the initial stages of deformation (e.g. $t<t_{\text{BN}}$, where t is time and $\;t_{\text{BN}}$ is a time before necking and will be specified later), the $|\varepsilon_{3}^{\text{RZ}}|$ and $|\varepsilon_{3}^{\text{BZ}}|$ values are equal because deformation is uniform. After the onset of localised necking (e.g. $t>t_{\text{AN}}$, where $t_{\text{AN}}$ is a time after necking and will be specified later), the deformation tends to localise within the necking band and thus, $|\varepsilon_{3}^{\text{BZ}}|$ becomes larger than $|\varepsilon_{3}^{\text{RZ}}|$. In the spatio-temporal method, two straight lines are fitted, for the data before $t_{\text{BN}}$ and after $t_{\text{AN}}$ respectively, using the least-squares method. The onset of localised necking is determined at the intersection of the two straight lines and the corresponding time is taken as the localised necking time $t_{\text{LN}}$, as demonstrated in Fig. 1(b). It should be noted that the straight line fitted using the data before $t_{\text{BN}}$, should in theory have a slope k = 1. The time immediately before fracture is taken as the fracture time $t_{F}$. Based on the times $t_{\text{LN}}$ and $t_{F}$, the corresponding limit strains (i.e. localised necking and fracture strains) are determined by using average strains within the zone BZ at the corresponding times.

### Theoretical basis

The theoretical basis of the spatio-temporal method for the determination of onset of localised necking can be demonstrated by analysing the relationship between the average thickness reductions within the zones RZ and BZ. In the initial stages of deformation, variations in the thickness reduction within the two zones are equal because the deformation is homogeneous and thus one has

(1) $$\Delta \left|\varepsilon_{3}^{\text{RZ}}\right|=\Delta \left|\varepsilon_{3}^{\text{BZ}}\right|$$

where $\Delta |\varepsilon_{3}^{\text{RZ}}|$ and $\Delta |\varepsilon_{3}^{\text{BZ}}|$ are the thickness reduction variations within the zones RZ and BZ, respectively. This indicates that before necking appears, the average thickness reductions within the zones RZ and BZ are equal, and a straight line with the slope k = 1 should fit the data.

After the onset of localised necking, deformation mainly occurs within the necking band and the thickness reduction variation within the zone RZ-I ($\Delta |\varepsilon_{3}^{\text{RZ}-I}|$) becomes very small, where the zone RZ-I is the zone RZ excluding the zone BZ. With further necking towards fracture, $\Delta |\varepsilon_{3}^{\text{RZ}-I}|$ will become zero. Hence, the variation of thickness reduction within the zone RZ can be expressed as

(2) $$\Delta \left|\varepsilon_{3}^{\text{RZ}}\right|=\frac{\Delta \left|\varepsilon_{3}^{\text{RZ}-I}\right|\times A_{\text{RZ}-I}+\Delta \left|\varepsilon_{3}^{\text{BZ}}\right|\times A_{\text{BZ}}}{A_{\text{RZ}-I}+A_{\text{BZ}}}=\Delta \left|\varepsilon_{3}^{\text{BZ}}\right|\times \frac{A_{\text{BZ}}}{A_{\text{RZ}-I}+A_{\text{BZ}}}$$

where $A_{\text{BZ}}$ and $A_{\text{RZ}-I}$ are the areas of the zones BZ and RZ-I, respectively.

With increasing deformation after localised necking, the area $A_{\text{BZ}}$ continues to increase, while the area $A_{\text{RZ}-I}$ changes little due to the small thickness reduction variation. Considering that the increase of the area $A_{\text{BZ}}$ is relatively small, there exists another approximately linear relationship between $\Delta |\varepsilon_{3}^{\text{RZ}}|$ and $\Delta |\varepsilon_{3}^{\text{BZ}}|$. Therefore, it is reasonable to fit another straight line by using the data after localised necking. The intersection of the two fitted straight lines can be taken as the onset of localised necking [11].

## Method procedure

In order to determine the limit strains at the onset of localised necking and fracture by using the novel spatio-temporal method, the following procedure should be followed:

• Step 1: Carry out the mechanical tests, and measure strain fields in the gauge area of the tested samples throughout deformation. During testing, the DIC method is employed to acquire the strain fields. For this purpose, random speckle patterns are prepared on sample surface and pictures are taken throughout deformation using cameras. In principle, the higher frame rate of the camera, the more accurate the strain measurement and the limit strain determination. Generally, the frame rate needs to be sufficient to give at least five frames between $t_{\text{AN}}$ and $t_{F}$.
• Step 2: Determine the location where fracture initiates, and the direction of the necking band. In the strain field immediately before fracture, the fracture initiation location is determined as the location where the thickness reduction or the major strain is the highest. Furthermore, the angle of the necking band, where the thickness reduction is localised, to one predefined specimen arm is determined.
• Step 3: Select the rectangular zones BZ and RZ at the fracture initiation location and obtain average strains within the two zones throughout deformation. In the strain field at the start of deformation, the zones BZ and RZ are selected at the determined fracture initiation location. All sides of the zones are either parallel or normal to the necking band direction, with initial lengths as recommended in Table 1. The lengths of the sides normal to the necking band are $W_{\text{BZ}}$ and $W_{\text{RZ}}$ in the zones BZ and RZ, respectively; The two zones have the same length $W_{L}$ of the sides parallel to the necking band. It should be noted that for the uniaxial tensile tests in which the necking band is at 45° to the tensile direction, the sides of lengths $W_{\text{BZ}}$ and $W_{\text{RZ}}$ should be parallel to the tensile direction, and accordingly, the sides of length $W_{L}$ are normal to the tensile direction. According to practical experience, the values of the factors a = b = c = 2 (Table 1) are especially recommended.

**Table 1:** Recommended values of parameters in the spatio-temporal method.

Parameters	Recommended values
$W_{\text{BZ}}$	*a*×*h,* 2 ≤ *a* ≤ 2.5
$W_{\text{RZ}}$	*b*×$W_{\text{BZ}}$*,* 2 ≤ *b*≤ 2.5
$W_{L}$	*c*×*h,* 1.5 ≤ *c* ≤ 3
$t_{\text{BN}}$	*f*×$t_{F}$*,* 0.3 ≤ *f* ≤ 0.6
$t_{\text{AN}}$	*g*×$t_{F}$*,* 0.99 ≤ *g* ≤ 0.998

* h is the initial thickness of material.
• Step 4: Determine the onsets of localised necking and fracture. On a plot of $|\varepsilon_{3}^{\text{RZ}}|$ against $|\varepsilon_{3}^{\text{BZ}}|$, two straight lines are fitted using the least square method for the experimental data in the ranges of $0\leq t\leq t_{\text{BN}}$ and $t_{\text{AN}}\leq t\leq t_{F}$, respectively. The fracture time $t_{F}$ is determined as the time immediately before fracture. Recommended values of the times $t_{\text{BN}}$ and $t_{\text{AN}}$ are given in Table 1. The onset of localised necking is taken to be located at the intersection of the two fitted straight, and the corresponding time is taken as the localised necking time $t_{\text{LN}}$. It should be noted that for the tensile tests under equi-biaxial tension, a large value for the factor g (e.g. g = 0.995) should be chosen due to the relatively fast development from localised necking to fracture in the condition. Based on experience thus far, the values of the factors f = 0.4, and g = 0.99 are especially recommended.
• Step 5: Determine the limit strains at the onsets of localised necking and fracture. Based on the localised necking and fracture times $t_{\text{LN}}$ and $t_{F}$ obtained in Step 4, the corresponding average strains within the zone BZ are taken as the localised necking and fracture strains, respectively.

## Method validation

### Application to uniaxial tensile tests

**Fig. 2:**
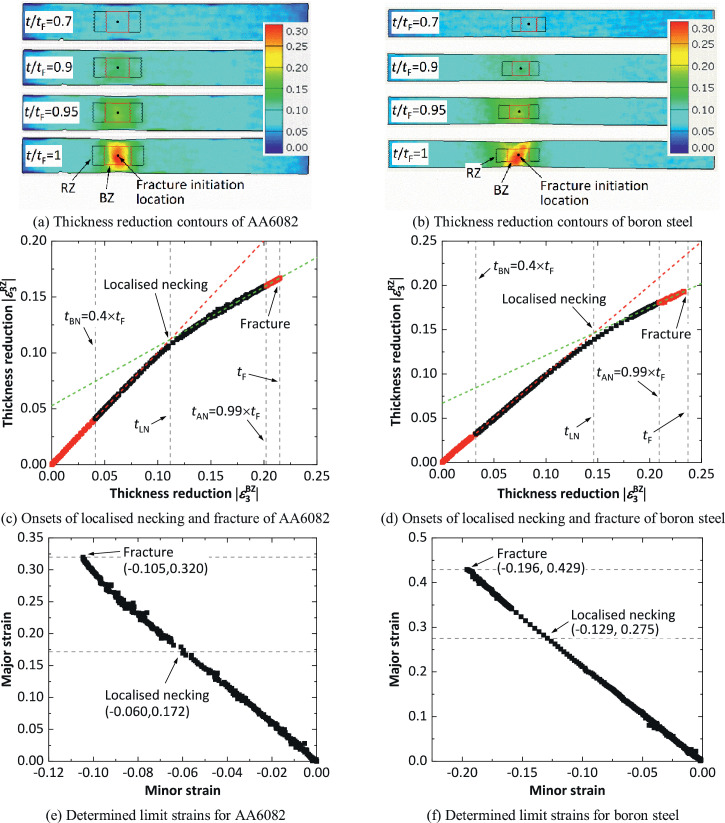
Determination of localised necking and fracture strains using the spatio-temporal method, for AA6082 and boron steel in the uniaxial tensile tests.

The spatio-temporal method has been validated by applying it to uniaxial tensile tests on two different sheet materials: AA6082 and boron steel. The two materials have the same thickness of 1.5 mm. The details of the uniaxial tensile tests including the geometry and dimensions of the dog-bone specimens have been given in Ref. [11]. Figs. 2(a) and 2(b) show the thickness reduction contours in the gauge area of the dog-bone specimens measured using DIC, at different normalised times $t/t_{F}$. In the strain contours immediately before fracture ($t/t_{F}$ = 1), the fracture initiation locations are determined, where the thickness reduction is the highest. Then in the strain contours at the start of deformation ($t/t_{F}$ = 0), the zones RZ and BZ are selected at the fracture initiation location, with initial side lengths set by choosing the factor values listed in Table 2. It can be seen in Figs. 2(a) and 2(b) that both the area and the shape of the zones RZ and BZ change during deformation. The average thickness reductions within the two zones are calculated throughout deformation. Figs. 2(c) and 2(d) show the determination of the onset of localised necking by plotting the thickness reduction within the zone RZ against that in BZ. The times $t_{\text{BN}}$ and $t_{\text{AN}}$ are determined by choosing the factor values listed in Table 2, i.e. $t_{\text{BN}}$ = 0.4$t_{F}$ and $t_{\text{AN}}$ = 0.99$t_{F}$. In Figs. 2(c) and (d), the two straight lines (dashed) fitted by using the experimental data (red points) in the time range of $0\leq t\leq t_{\text{BN}}$ and $t_{\text{AN}}\leq t\leq$$t_{F}$, respectively, can be seen. The number of the red points during $t_{\text{AN}}\leq t\leq$$t_{F}$ in Figs. 2(c) and 2(d) are 21 and 53, respectively, which are sufficient to ensure the accuracy of the fitted straight line. Importantly, the onset of localised necking ($t_{\text{LN}}$) is determined by finding the intersection of the two lines. Indeed, the experiment data of the thickness reduction before and after $t_{\text{LN}}$ exhibits two approximately linear relationships, and most of them lie on or close to the corresponding one of the two fitted straight lines. Based on the determined onsets of localised necking and fracture, the corresponding limit strains are determined by using the average strains within the zone BZ, as presented in Figs. 2(e) and 2(f). For AA6082 in the uniaxial tensile tests, the localised necking and fracture strains obtained were (−0.06, 0.172) and (−0.105, 0.32) respectively, while for boron steel, they were (−0.129, 0.275) and (−0.196, 0.429), where the first element in each pair of values refers to the minor strain and the second element to the major strain.

**Table 2:** Factor values chosen for limit strain determination using the novel spatio-temporal method.

*a*	*b*	*c*	*f*	*g*
2	2	2	0.4	0.99

### Should zone RZ exclude zone BZ?

**Fig. 3:**
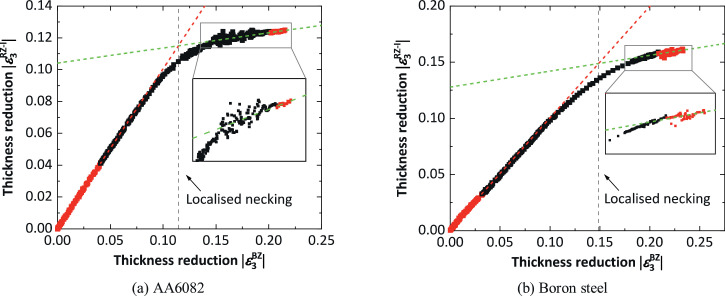
Determination of onset of localised necking by plotting the average thickness reduction within the zone BZ and the zone RZ-I which excludes the zone BZ, for AA6082 and boron steel in the uniaxial tensile tests.

One may assume that the zone RZ should exclude the zone BZ in the determination of the onset of localised necking, i.e. the average thickness reduction within the zone RZ-I rather than the zone RZ should be adopted for the straight line fitting. This is because $\Delta |\varepsilon_{3}^{\text{RZ}-I}|$ changes to zero after the localised necking, leading to a relatively large change of slope between the fitted straight lines and making the determination of the onset of localised necking easier. However, the straight line fitted using the experimental data after the time $t_{\text{AN}}$ may depend significantly on the $t_{\text{AN}}$ value chosen, i.e. the onset of localised necking determined using this method maybe significantly dependant on the $t_{\text{AN}}$ value. To demonstrate this, the thickness reductions within the zones RZ-I and BZ for the determination of the onset of localised necking in the uniaxial tensile tests, are plotted in Figs. 3(a) and 3(b) for AA6082 and boron steel, respectively. The two straight lines are fitted using the experimental data (red points) in the same time ranges as in Fig. 2. Indeed, compared with the results in Figs. 2(c) and 2(d), in which the zone RZ includes the zone BZ, a larger change of the slopes between the two fitted straight lines can be seen. In addition, the values of the thickness reduction within the zone BZ at the determined onset of localised necking are almost the same either including or excluding the zone BZ, for the two materials separately. However, the experimental data after the determined onset of localised necking do not exhibit a good linear relationship. This is different from the results shown in Figs. 2(c) and 2(d), in which most of the experimental data after the determined onset of localised necking are well fitted by a linear relationship. Therefore, in order to enable stable determination of the limit strains, the zone RZ should include the zone BZ for the determination of the onset of localised necking using the spatio-temporal method.

### Comparison with existing methods

**Fig. 4:**
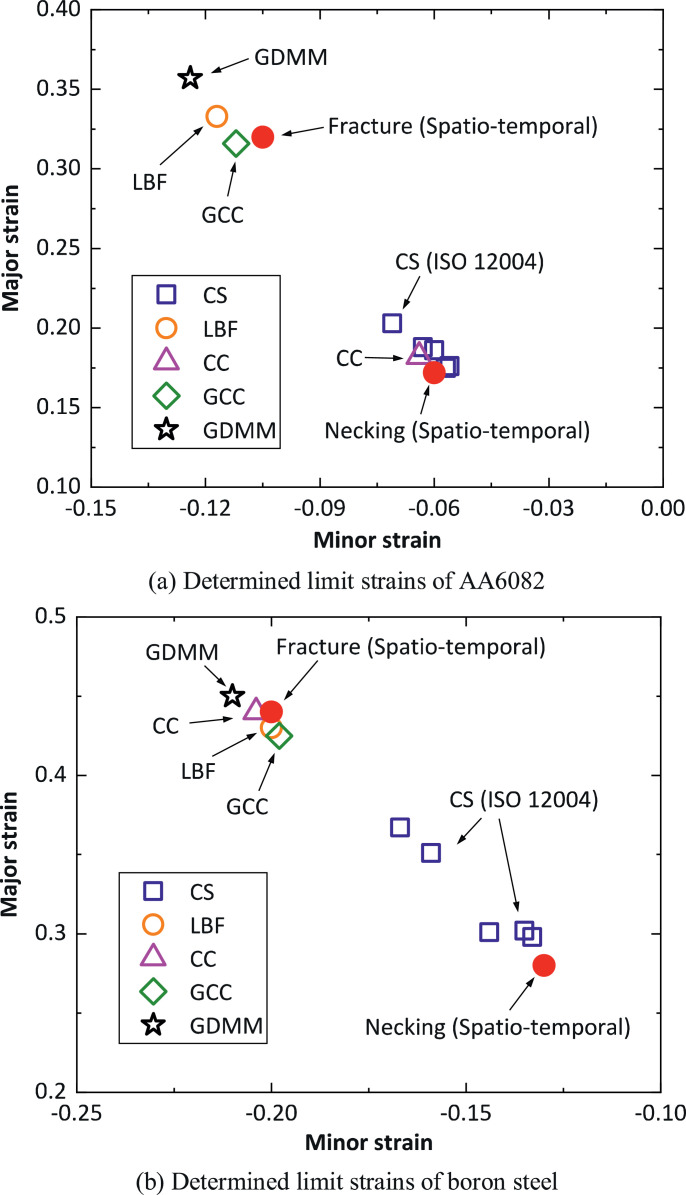
Comparison of the limit strains determined using the various methods including the spatio-temporal method for 1.5 mm thick AA6082 and boron steel in the uniaxial tensile tests, CS: cross-section method, LBF: linear best fit method, CC: correlation coefficient method, GCC: gliding correlation coefficient method, GDMM: gliding difference of mean to median method.

The spatio-temporal method is compared with several other existing methods with regard to the limit strains determined at the onset of localised necking. The existing methods include: the cross-section (CS) method in the standard ISO 12004–2:2008 [1], the linear best fit method (LBF) [8,10], the correlation coefficient (CC) method [8,12], the gliding correlation coefficient (GCC) method [8], and the gliding difference of mean to median (GDMM) method [8]. The details of how the limit strain is determined using these methods have been given in Ref. [11]. Fig. 4(a) shows a comparison of the limit strains determined, plotted on axes of major and minor strains, for AA6082 in uniaxial tensile tests and 4(b) is the corresponding plot for boron steel. In each of the tests, the limit strains determined using the different methods have an approximately identical ratio of major strain to minor strain, so that only the major strains determined using the different methods are compared. In Fig. 4(a) for AA6082, the spatio-temporal method gives a major strain of 0.172 at the onset of localised necking, which is very close to the values of 0.185 (average of the five limit strain points) and 0.175 determined using the CS and CC methods, respectively. However, the major strains determined using the LBF, GCC and GDMM methods are at least 71% higher than 0.185, and even a little higher than the major strain determined at fracture using the spatio-temporal method. In Fig. 4(b) for boron steel, a large scatter can be observed amongst the major strains determined using the CS method, and two major strain values are at least 17% higher than the other three values. This is due to the difference of the measured strain distribution along the predefined cross-sections, resulting in the two unreliable higher limit strain points in the use of the CS method. Importantly, the spatio-temporal method determines the major strain to be 0.28 at the onset of localised necking, which is only 6.6% smaller than 0.30, the average of the three lower limit strain points obtained using the CS method. In addition, the major strains determined using the LBF, CC, GCC, and GDMM methods are very close to each other, and also close to the major strain at fracture obtained using the spatio-temporal method. In summary, the comparison as presented in Figs. 4(a) and 4(b) indicates that the spatio-temporal method determines almost the same limit strain as that obtained using the CS method, while the other methods like the LBF and GDMM methods, may overestimate the limit strain at the onset of localised necking.

### Application to equi-biaxial tensile test

**Fig. 5:**
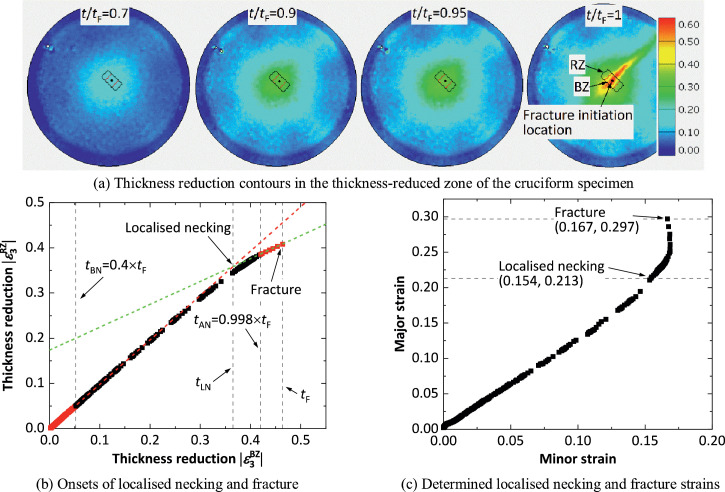
Determination of the localised necking and fracture strains by using the spatio-temporal method for 1.5 mm thick AA5754 sheet in the equi-biaxial tensile test with cruciform specimens.

The spatio-temporal method has also been applied to equi-biaxial tensile tests on 1.5 mm thick AA5754 cruciform specimens. The experimental details including setup and tensile speed have been given in Ref. [11]. When using the DIC technique for strain measurement, a frame rate of 125 frames per second (fps), which can be easily achieved, was chosen to generate sufficient data points throughout deformation. Furthermore, both the geometry and dimensions of the cruciform specimens have been investigated and optimised in Ref. [13], to enable production of a proportional equi-biaxial strain path at the fracture initiation location. Fig. 5(a) shows the thickness reduction contours within the central thickness-reduced zone in the cruciform specimen at different normalised times. In the strain contour at $t/t_{F}$ = 1, the fracture initiates near the specimen centre, where the thickness reduction is the highest. The necking band is orientated at about 45° to the arm direction. Given that the initial thickness near the centre is about 0.5 mm, both zones RZ and BZ are selected in the strain contour at the start of deformation, with initial dimensions $W_{\text{BZ}}$ = 1 mm (a = 2), $W_{\text{RZ}}$ = 2 mm (b = 2) and $W_{L}$ = 1 mm (c = 2). Then the onset of localised necking is determined by plotting the thickness reduction in RZ against that in BZ, as presented in Fig. 5(b), in which the two dashed straight lines are obtained by fitting to the red points in the time ranges of $0<t\leq 0.4t_{F}$ (f = 0.4) and 0.998$t_{F}<t\leq t_{F}$ (g = 0.998), respectively. The reason for choosing g = 0.998, which is different from the value of 0.99 adopted in the uniaxial tensile tests, is that the transition from localised necking to fracture under equi-biaxial tension is much quicker than that under uniaxial tension. Fig. 5(c) shows the strain path in the zone BZ and the localised necking and fracture strains obtained based on the onsets of the localised necking and fracture determined. The necking strain for 1.5 mm thick AA5754 sheet in the equi-biaxial tensile test is (0.154, 0.213) and the fracture strain is (0.167, 0.297). The strain path is almost linear until the occurrence of the localised necking, after which it deviates from linearity because the minor strain increases more slowly than the major strain.

### Repeatability for limit strain determination

**Fig. 6:**
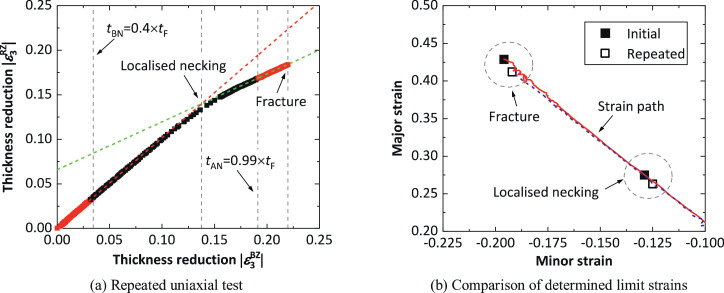
Limit strain determination in a repeated uniaxial test on boron steel using the spatio-temporal method, and comparison of the determined limit strains from the initial (Fig. 2(f)) and repeated uniaxial tests.

In order to demonstrate the repeatability of the spatio-temporal method with respect to the limit strain determination, the uniaxial test (Fig. 2) on boron steel sheet was repeated and the determined limit strains from the two tests, using the spatio-temporal method, were compared. Fig. 6(a) shows the determined onsets of localised necking and fracture in the repeated uniaxial test. Similar to the results shown in Fig. 2(d), experimental data of thickness strain within the zone RZ against that within the zone BZ exhibits two linear relationships which are well presented by the fitted dash lines. Fig. 6(b) shows the determined limit strains at the onsets of localised necking and fracture from the two tests, together with corresponding strain paths. The strain paths in the two tests are almost same, and importantly, the difference between the determined limit strains at either localised necking or fracture is less than 5%. This demonstrates that the spatio-temporal method has a good repeatability for the determination of the limit strains.

**Fig. 7:**
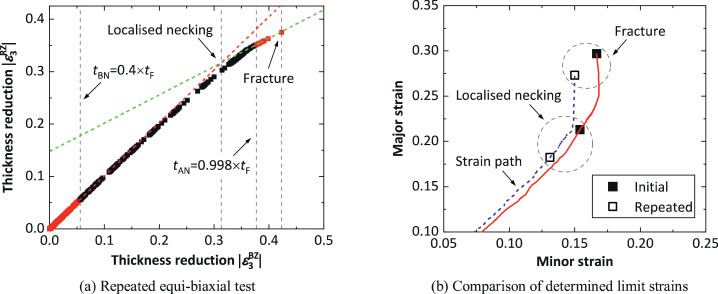
Limit strain determination in a repeated equi-biaxial test on AA5754 using the spatio-temporal method, and comparison of the determined limit strains from the initial (Fig. 5(c)) and repeated biaxial tests.

The equi-biaxial test (Fig. 5) on AA5754 sheet was also repeated, and the limit strains were determined using the spatio-temporal method. Fig. 7(a) shows the determined onsets of localised necking and fracture, in which 6 points after $t_{\text{AN}}$ were available to fit the straight line, similar to the results presented in Fig. 5(b). Fig. 7(b) shows the determined limit strains from the two biaxial tests, together with corresponding strain paths. The determined limit strain values at either localised necking or fracture from the two tests are slightly different. For example, the determined major strain at fracture from the repeated test is about 8% lower than that from the initial test. This may be attributed to the divergency of the strain paths at the later stages of deformation. The results in Fig. 7 indicates that the spatio-temporal method has a good repeatability for applying to equi-biaxial tests.

## Summary

A spatio-temporal method has been developed for determination of necking and fracture strains, which are necessary to construct forming limit diagrams and fracture forming limit diagrams. In this method, the onset of localised necking is determined by analysing average thickness reduction within preselected zones around the location where fracture initiates. It has been validated that the method has stable ability to determine limit strains in either uniaxial tests using dog-bone specimens, or biaxial tests using cruciform specimens. Moreover, in the uniaxial tests, the method can reproduce results from the cross-section method which has been widely accepted. The method can be easily reproduced by following the procedure and recommended parameters.

Compared with the cross-section method in the standard ISO 12004:2, the spatio-temporal method can be applied to cruciform specimens which usually have a relatively small gauge area. Furthermore, using the spatio-temporal method enables the determination of the limit strains at onset of necking and fracture in one process, which are necessary to construct the FLDs and FFLDs, respectively.

## Declaration of Competing Interest

The authors declare that they have no known competing financial interests or personal relationships that could have appeared to influence the work reported in this paper.
